# Construction and verification of rehabilitation nursing program for shoulder and neck discomfort after thyroid cancer surgery: A pilot randomized controlled trial

**DOI:** 10.1097/MD.0000000000039291

**Published:** 2024-08-16

**Authors:** Qiuqin Xu, Hongzhan Jiang, Yuanchan Li, Xiushan Qi, Lijuan Chen

**Affiliations:** a Xiamen Hospital, Beijing University of Chinese Medicine, Xiamen, China; b Xiamen TCM Hospital Affiliated to Fujian University of Traditional Chinese Medicine, Xiamen, China; c School of Nursing, Beijing University of Chinese Medicine, Beijing, China; d Zunyi Hospital of Traditional Chinese Medicine, Zunyi, China; e Department of General Surgery, Zhongshan Hospital of Xiamen University, School of Medicine, Xiamen University, Xiamen, China.

**Keywords:** empowerment theory, postoperative, rehabilitation, shoulder and neck discomfort, thyroid cancer

## Abstract

**Background:**

To develop a nursing program for the prevention and rehabilitation of shoulder and neck discomfort after thyroid cancer surgery based on the empowerment theory, and to evaluate the application effect of the program.

**Methods:**

The prevention and rehabilitation nursing program for shoulder and neck discomfort after thyroid cancer surgery was established by literature review and the Delphi method. Between July 2022 and January 2023, a total of 62 postoperative thyroid cancer patients were recruited and randomly allocated to either the intervention group (n = 31) or the control group (n = 31) in this randomized controlled trial. Comparisons of shoulder and neck function, self-efficacy, and quality of life between the 2 groups were performed using a 2-sample independent *t* test, Wilcoxon rank-sum test, and repeated-measures analysis of variance.

**Results:**

At the end of the study, the control group and intervention group were 30 cases each completed the study. After the intervention, the self-efficacy score of the intervention group was higher than the control group (*P *< .05), and the score of emotional function, cognitive function, and overall health dimension of the intervention group was higher than the control group (*P* < .05). The pain dimension score of the intervention group was lower than the control group (*P* < .05). There were significant differences in the group and time effects of the total shoulder joint scores between the 2 groups (*P* < .05).

**Conclusion:**

This study demonstrated that the shoulder and neck rehabilitation nursing program can alleviate the symptoms of shoulder and neck discomfort and improve patients’ self-efficacy and quality of life.

## 1. Introduction

Thyroid cancer (TC) represents the predominant malignancy observed within the head and neck region. TC ranks 7th in terms of newly diagnosed tumors in China each year, with over 220,000 instances in 2020.^[1]^ It can be categorized into 3 main pathological subtypes: differentiated TC, anaplastic TC, and medullary TC. Notably, differentiated TC has a usually good prognosis, and accounts for over 95% of all diagnosed cases of TC.^[2]^ Surgical resection remains the cornerstone of TC treatment, offering remarkable efficacy, high cure rates, and favorable prognosis.^[3]^

However, the procedure and associated psychological factors often lead to various physical and mental complications, such as shoulder and neck discomfort, which can impede patient recovery. Shoulder and neck discomfort, characterized by pain, pulling, distension, contraction, and compression, affects up to 44.6% of patients following TC surgery, 28.9% of patients may experience persistent neck numbness, and 26.8% may suffer from limited shoulder or neck mobility, even over 1 year after treatment.^[4]^ Shoulder and neck discomfort significantly impairs the quality of life for TC patients post-surgery, and if left unaddressed, can result in deteriorating shoulder stability and functional loss.^[5]^ Therefore, shoulder and neck rehabilitation is the focus of postoperative TC care. Previous studies have demonstrated that effective preventive or rehabilitative interventions can significantly alleviate shoulder and neck discomfort in patients.^[6]^ Currently, researchers are exploring various approaches, including postural exercises, postural education, myofascial release, and joint mobilization techniques, to improve rehabilitation outcomes.^[7]^

Additionally, TC patients often exhibit poor self-efficacy and low enthusiasm for rehabilitation due to surgical stress and other effects.^[8]^ However, the current postoperative rehabilitation protocols for shoulder and neck regions following TC surgery exhibit several limitations. These protocols lack a comprehensive approach, failing to adequately address patients’ cognitive understanding, rehabilitation needs, and active participation. Furthermore, the research efforts in this domain have been relatively narrow in scope, neglecting the multifaceted nature of the rehabilitation process. Consequently, there is a critical need to establish a comprehensive, systematic, and scientifically grounded prevention and rehabilitation nursing program.

This study included postoperative patients with TC, developed and implemented a nursing plan for the prevention and rehabilitation of shoulder and neck discomfort after TC surgery, grounded in the empowerment theory, through a literature review and Delphi expert consultation. The program aimed to improve patient self-efficacy, shoulder and neck function, and quality of life.

## 2. Materials and methods

### 2.1. Study participants

This randomized controlled trial enrolled 62 patients who underwent thyroidectomy at the Department of General Surgery in Zhongshan Hospital Xiamen University between July 2022 and January 2023. According to the order of admission, patients were randomly divided into intervention (n = 31) and control (n = 31) groups by using the random number table method. The sample size was calculated using the formula as follows:


$$\begin{array}{l} n=2\times {\left\{\left(Z_{\alpha }+Z_{\beta }\right)\times \sigma /\delta \right\}}^{2} \end{array}$$

**Figure 1.**
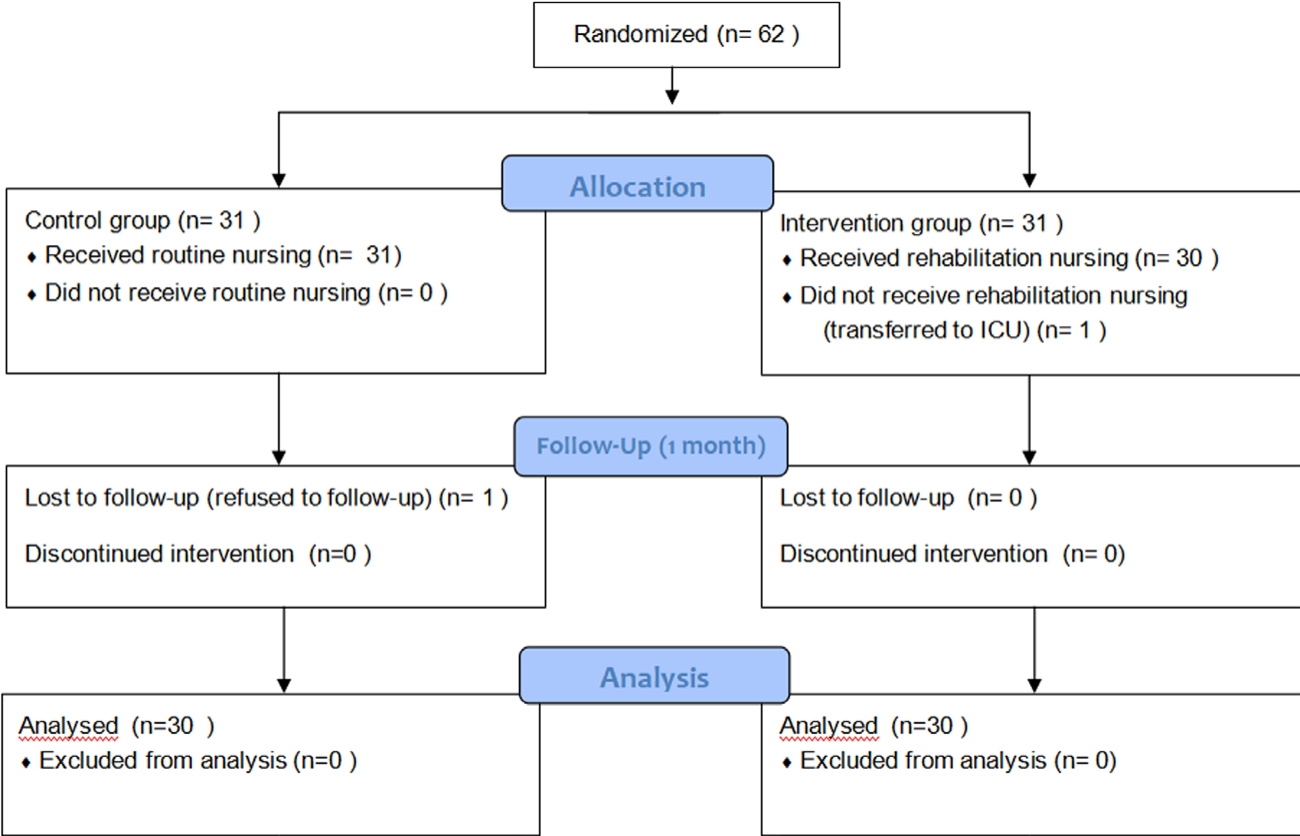
Flowchart of research process.

Based on a previous study^[9]^ using 95% confidence intervals (*α* = 0.05, *Z*_*α*_ = 1.96), 90% power (*β* = 0.1, *Z*_*β*_ = 1.282), and a mean value of the Constant–Murley Score (CMS) outcome measure of 95.22 (4.90) for the intervention group and 84.89 (9.26) for the control group (*σ* = 9.26, *δ* = μ_1_-μ_2_ = 10.33). To account for a 20% attrition rate, a total of 62 patients were ultimately enrolled, with 31 patients randomly assigned to each group (Fig. 1).

The inclusion criteria were as follows: meeting the diagnostic criteria for TC; age 18 and above; mentally sound, with clear consciousness and normal communication skills; those who signed the informed consent form, and agreed to participate.

The exclusion criteria were as follows: shoulder and neck injuries caused by disease or other operations (e.g., cervical spondylosis, cervical spinal cord injury, neck surgery); impaired function of vital organs, such as the heart, brain, kidney, or liver; participation in other clinical trials that could affect the evaluation of the study’s intervention.

This study was approved by the ethical committee of the Zhongshan Hospital Xiamen University (approval number: xmzsyyky2021-045).

### 2.2. Research methods

The control group was provided with routine nursing, which informed patients about operation-related knowledge, and required patients to follow the doctor’s advice. The intervention group received the rehabilitation nursing plan developed in the earlier stage of this study. The intervention process was as follows:

#### 2.2.1. Development of a rehabilitation nursing program

Literature review and Expert Delphi Consultation were used to develop a shoulder and neck discomfort rehabilitation nursing program. The process was as follows:

1. Establishment of the research team. The research group was composed of the following members: 2 chief physicians, 1 chief nurse, 2 rehabilitation therapists, 3 nurses in charge, 6 graduate students. All members of the research team underwent systematic training and participated in the following tasks: literature review and retrieval, preparation of the intervention program content, selection of experts for the Delphi consultation, and data collection and analysis.
2. Theoretical framework. The theoretical foundation of this study is the empowerment theory, which originated from social movements in the late 1960s and 1970s and was introduced into disease management in the early 1990s.^[10]^ Rappaport^[11]^ defined empowerment as a framework that links individual strengths and abilities, natural support systems, and proactive behaviors to social policy and change. In the nursing domain, the empowerment approach enables individuals to identify, promote, and enhance their capacity to address needs and solve problems, while also utilizing the necessary resources to actively participate in health management.^[12,13]^ This study was guided by the implementation steps of the empowerment education model, which include problem identification, emotion catharsis, goal setting, plan confirmation, and effect evaluation. These elements were integrated with the content of shoulder and neck rehabilitation nursing for patients after TC surgery, forming the basis for developing the prevention and rehabilitation nursing plan.
3. Literature review. “thyroid cancer/thyroid malignancy/thyroid malignancy,” “shoulder and neck discomfort/neck and shoulder discomfort/neck and shoulder syndrome/shoulder and arms syndrome/neck edema/postural syndrome/neck muscle strain/shoulder and neck/shoulder/neck,” “rehabilitation/nursing/intervention/sports/training/functional exercise/psychological/psychological care/emotional/postural/postural care” were used as search terms to search CNKI, Wanfang, VIP, PubMed, Cochrane Library, Web of Science and the search period ranged from the establishment of each database to January 2023. Through the analysis of the included literature, the research team extracted relevant content on postoperative shoulder and neck care for TC patients. This informed the development of the preliminary rehabilitation nursing plan.
4. Preparation of an expert questionnaire. Based on the results of the group discussion and literature review, the research team developed a preliminary correspondence questionnaire for the nursing plan. The content of the questionnaire mainly includes 3 parts: the preface, the questionnaire of experts’ situations, and the evaluation content of the rehabilitation nursing scheme. The preface mainly introduces the research background, purpose and significance of this study, and the method of filling in the form. The questionnaire on experts’ situation includes the basic information table of experts, the familiarity table, and the questionnaire based on experts’ judgment on the contents of letters and inquiries. The evaluation content of the scheme is divided into 1, 2, and 3 levels of indicators, and the Likert 5-level scoring method is used for the importance of each level of indicators. Experts can indicate the modification opinions or reasons for the corresponding indicators in the column “Modification Opinions,” or add the indicators they think should be added in the column “Suggested Indicators.”
5. Expert selection. For this study, 16 experts were selected for 2 rounds of expert letter consultations. The experts were drawn from thyroid diagnosis and treatment, rehabilitation, and clinical nursing. The inclusion criteria were as follows: bachelor’s degree or higher; minimum of 10 years of relevant work experience; employed at a tertiary hospital; provided informed consent and participated voluntarily. All selected experts met these criteria and actively engaged in the consultation process.
6. Delphi consultation implementation. In this study, the questionnaires were distributed to the experts via email or WeChat. After the first round of expert consultation, the collected data were collated and analyzed. Based on the index screening criteria and expert feedback, a second round of questionnaires was developed and the expert consultation was conducted again. The interval between each round of correspondence was 2 to 3 weeks. The consultation process continued until the expert opinions converged. The inclusion criteria for each index were: average score >4.00 and coefficient of variation <0.25. Two rounds of Delphi consultation were carried out in this study. In the first round, 16 expert consultation questionnaires were distributed, with 15 recovered (93.75% response rate), 11 experts provided suggestions and guidance, expert familiarity was 0.89, judgment basis was 0.93, and expert authority coefficient was 0.91. The coefficient of variation for the importance scores ranged from 0.00 to 0.39, and Kendall W was 0.239 (*P* < .05).
7. Implementation of the rehabilitation nursing program. According to the preestablished program content, the research team conducted the following interventions:

Baseline assessments were conducted within 24 hours of admission using the following instruments: the Strategies Used by Patients to Promote Health (SUPPH) scale, the Quality-of-Life Questionnaire Core-30 (QLQ-C30), and the CMS. These assessments evaluated patients’ educational attainment, disease comprehension, psychological well-being, self-efficacy, shoulder and neck functionality, activities of daily living, and overall quality of life. Patients were encouraged to express any questions or concerns regarding their TC diagnosis and received timely feedback and guidance on relaxation techniques and relevant preoperative precautions. Upon obtaining informed consent, patients or their designated family members were enrolled in a dedicated WeChat group for communication and support.

On the day of surgery, 2 research nurses provided emotional support and addressed patient concerns. Patients received a copy of the “Manual of Shoulder and Neck Function Exercise after Thyroid Cancer Surgery.” Family members were instructed on their role in postoperative care, specifically regarding postural adjustments. Upon return to the ward, patients were positioned supine with a soft pillow supporting their heads. Family members were advised to elevate the head of the bed by approximately 30° every 1 to 2 hours, to a maximum incline of 60°. Once vital signs stabilized, patients were encouraged to gargle, with assistance to maintain a lateral neck angle within 30° for 5 to 10 minutes, 1 to 2 times daily. Positive reinforcement was provided for patient efforts. The research team, comprising physicians and rehabilitation specialists, conducted real-time assessments of patients’ exercise capacity, considering factors such as postoperative bleeding and pain levels. Exercise regimens were dynamically adjusted based on individual patient needs under the supervision of the head nurse.

On postoperative day 1, research nurses assessed patients’ shoulder and neck function using a goniometer and the CMS. Patients were interviewed regarding their subjective experiences with the gargling exercises, including any difficulties or discomfort. Open-ended questions were utilized to explore barriers to exercise adherence (e.g., “Do you experience any discomfort while gargling?” or “What concerns you about performing the gargling exercises?”). Patients received education regarding the rationale for the exercises and the potential consequences of nonadherence. Exercise technique was evaluated, and subsequent training plans were tailored based on individual patient progress. Shoulder and neck recovery was assessed collaboratively by the physician and rehabilitation therapist, informing adjustments to the rehabilitation plan. Patients received guidance from research nurses on progressively increasing their lateral neck range of motion during gargling exercises, aiming for a range of 30° to 60° degrees. Patients were also instructed on incorporating head-up movements (within 30°) during gargling, for 5 to 10 minutes, 1 to 2 times daily. Additionally, patients were instructed on upper limb exercises, including lateral raises, forward flexion (60° relative to the torso), and backward extension (30° relative to the torso), for 5 minutes, 3 times daily. The head nurse provided support and supervised all exercises.

From postoperative days 4 to 7, daily assessments of patient progress were conducted by physicians and rehabilitation therapists. Research nurses facilitated patient self-evaluation of their rehabilitation progress and guided gradually increasing the range of motion for head lifting and gargling exercises. Patients were instructed to perform head lifts and neck extensions within a range of 30° to 60°, alternating between these movements. Upper limb exercises during this phase emphasized wall climbing with a 45° to 60° inclination. Shoulder and neck exercises were progressively incorporated and included shoulder shrugs, head turns (up and down), and ear-to-shoulder stretches (angle <30°) for 10 minutes, 3 times daily. Research nurses provided ongoing support by addressing patient questions and concerns. The remaining 2 research nurses maintained meticulous records of patient rehabilitation data, which were reviewed daily by the head nurse.

Upon discharge (1 week postoperatively), research nurses evaluated patients’ shoulder and neck function using the CMS. Patients were queried about any challenges encountered during their rehabilitation and provided with appropriate guidance. Patient self-assessment of rehabilitation progress was also encouraged. A personalized follow-up rehabilitation plan was developed by the rehabilitation therapist and attending physician, taking into consideration the patient’s postoperative recovery. To facilitate ongoing support, educational materials, and instructional videos were created by the research team. These resources, covering shoulder and neck exercises to be performed during the first postoperative month, were disseminated to patients and their families via WeChat. Exercises included combined shoulder and neck movements (e.g., combing hair, shoulder pronation, and supination) for 15 minutes, 3 times daily. Patients were encouraged to establish reminders for their exercises and family members were enlisted to provide support and supervision. Patients and their families were also instructed to provide regular feedback on the patient’s progress. Furthermore, the research team utilized WeChat and telephone calls to encourage patient engagement in enjoyable and relaxing activities to mitigate negative emotions. Twice-weekly follow-up via WeChat was conducted to monitor shoulder and neck function, wound healing, pain levels, medication adherence, and psychological well-being. Throughout the entire process, the head nurse maintained real-time oversight of the research progress and provided dynamic management.

At the 1-month postoperative follow-up appointment, research nurses reassessed patients’ shoulder and neck function, self-efficacy, and quality of life utilizing the CMS, SUPPH scale, and QLQ-C30, respectively. The follow-up assessment mirrored the baseline assessment in terms of content and delivery. Patients engaged in self-evaluation of their progress and a personalized long-term rehabilitation plan was collaboratively established with the rehabilitation team, physicians, and nurses. Ongoing support and monitoring were provided by the research team through WeChat or telephone follow-up, tailored to individual patient needs.

This study employed a noninvasive intervention strategy, serving as an adjunctive rehabilitation approach for patients with TC and posing minimal risk to their health and safety. Stringent measures were implemented to safeguard patient privacy and confidentiality. All interventions were initiated following informed consent, and patients retained the right to withdraw from the study at any point should they experience any discomfort or adverse effects.

### 2.3. Research tools

The shoulder and neck function was assessed using the Chinese version of the CMS at 4-time points: preoperatively, on the first postoperative day, at 1 week postoperatively, and 1 month postoperatively. The CMS,^[14]^ developed by Constant and Murley, is a widely employed evaluation system endorsed by the European Shoulder Joint Evaluation Association. After the Chinese translations by Yao et al,^[15]^ Cronbach α coefficient of the scale was determined to be 0.981, indicating a high level of internal consistency. Furthermore, the test-retest reliability was found to be 0.827, demonstrating robust temporal stability, thereby ensuring its validity and consistency in measuring shoulder and neck function.^[15]^

The Chinese version of the SUPPH scale was employed to assess patients’ self-efficacy both pre- and post-intervention. The SUPPH scale, originally developed by Lev and Owen,^[16]^ demonstrated strong reliability and validity in its Chinese translation by Yuan et al (2015). Cronbach α coefficients for the translated scale ranged from 0.849 to 0.970, indicating its suitability for assessing self-management efficacy among cancer patients in China.^[16,17]^

The Chinese version of the QLQ-C30 was utilized to assess the quality of life of patients with TC both pre- and post-intervention. This scale, with a Cronbach α coefficient ranging from 0.75 to 0.91, has demonstrated good reliability and validity, thereby ensuring its accuracy in measuring the quality of life of patients with TC.^[18]^

### 2.4. Statistical analysis

Statistical analysis was performed using SPSS 23.0 software. Normally distributed measurement data were expressed as mean ± standard deviation ($\bar{x}$ ± *s*), and comparisons between groups were conducted using 2-sample independent *t* tests. Non-normally distributed data were represented as medians and interquartile ranges and analyzed using the Wilcoxon rank-sum test. Categorical data were expressed as frequency and analyzed using the chi-squared test or Fisher exact test, as appropriate. Ordinal data were expressed as composition ratios and analyzed using the Wilcoxon rank-sum test. A significance level of *P* < .05 was used to determine statistical significance.

## 3. Results

At the end of the study, 2 cases were lost to follow-up, comprising 1 control group participant who declined postoperative follow-up and 1 intervention group participant who was transferred to the intensive care unit due to sudden postoperative complications. Ultimately, a total of 60 patients were included in the analysis, with 30 patients in the control group and 30 patients in the intervention group. Baseline demographic data revealed no significant differences between the 2 groups (*P* > .05) (Table 1).

**Table 1:** Intergroup comparison of baseline characteristics between 2 groups [n(%)/$\bar{x}$ ± *s*].

Objectives	Categorization	Control group	Observation group	χ^2^/*t*/*Z*	*P*
Age (yr)	—	39.57 ± 1.14	42.93 ± 1.39	1.717*	.064
Sex	Male	9 (30.00)	9 (30.00)	0.000†	1.000
	Female	21 (70.00)	21 (70.00)		
Educational attainment	Primary and below	3 (10.00)	4 (13.33)	2.893‡	.716
	Lower secondary education	3 (10.00)	6 (20.00)		
	Upper secondary education	2 (6.67)	4 (13.33)		
	Postsecondary education	8 (26.67)	5 (16.67)		
	Undergraduate degree	13 (43.33)	10 (33.33)		
	Postgraduate degree	1 (3.33)	1 (3.33)		
Marital status	Single status	7 (23.33)	1 (3.33)	6.010†	.111
	Married	22 (73.33)	27 (90.00)		
	Divorced	1 (3.33)	1 (3.33)		
	Widowed	0	1 (3.33)		
Faith	Yes	11 (36.67)	9 (30.00)	0.075†	.784
	No	19 (63.33)	21 (70.00)		
Residential area	Countryside	5 (16.67)	5 (16.67)	0.000†	1.000
	Town	25 (83.33)	25 (83.33)		
Occupation	Worker	0	3 (10.00)	9.198†	.239
	Healthcare professionals	1 (3.33)	3 (10.00)		
	Teacher	1 (3.33)	2 (6.61)		
	Entrepreneur	2 (6.67)	1 (3.33)		
	Salaried worker	14 (46.67)	11 (36.67)		
	Professionals	6 (20.00)	1 (3.33)		
	Retire	2 (6.67)	3 (10.00)		
	Other	4 (13.33)	6 (20.00)		
Medical coverage	Yes	28 (93.33)	29 (96.67)	0.000†	1.000
	No	2 (6.67)	1 (3.33)		

* *t*-statistic.

† Chi-squared value.

‡ Z-score.

### 3.1. Comparison of shoulder and neck function between the 2 groups

Before the intervention, no significant differences were observed between the 2 groups (*P* > .05). However, on the first postoperative day, the intervention group demonstrated significantly higher scores in pain, daily living ability, and total score compared to the control group (*P* < .05). At 1 week postoperatively, the total score of the intervention group remained significantly higher than that of the control group (*P* < .05). Furthermore, at 1 month postoperatively, the total score of the intervention group continued to be significantly higher than that of the control group (*P* < .05) (Table 2).

**Table 2:** Intergroup comparison of shoulder and neck functional scores between 2 groups [M (P25, P75)/$\bar{x}$ ± *s*].

Item/group	Pre-intervention	Postoperative day 1	1-wk postoperative follow-up	1-mo postoperative assessment
Pain				
Control group	15.00 (15.00–15.00)	5.00 (5.00–10.00)	10.00 (10.00–10.00)	15.00 (13.75–15.00)
Observation group	15.00 (15.00–15.00)	10.00 (5.00–10.00)	10.00 (10.00–15.00)	15.00 (15.00–15.00)
*t*/Z	0.463*	2.149*	1.787*	1.439*
*P*	.643	.032	.074	.150
ADL				
Control group	40.00 (40.00–40.00)	12.00 (10.00–12.00)	12.00 (12.00–16.00)	16.00 (16.00–20.00)
Observation group	20.00 (20.00–20.00)	12.00 (12.00–12.00)	12.00 (12.00–16.00)	16.00 (16.00–20.00)
*t*/Z	0.464*	2.629*	0.676*	0.152*
*P*	.642	.009	.499	.879
ROM				
Control group	20.00 (20.00–20.00)	30.00 (26.00–32.00)	31.40 ± 5.23	36.00 (34.00–38.00)
Observation group	20.00 (20.00–20.00)	31.00 (30.00–34.00)	33.27 ± 3.69	38.00 (35.50–38.00)
*t*/Z	0.866*	1.414*	4.219†	0.650*
*P*	.386	.157	.116	.516
Muscular strength				
Control group	25.00 (25.00–25.00)	20.00 (20.00–25.00)	25.00 (20.00–25.00)	25.00 (20.00–25.00)
Observation group	25.00 (25.00–25.00)	20.00 (20.00–25.00)	25.00 (20.00–25.00)	25.00 (20.00–25.00)
*t*/Z	0.463*	1.712*	0.366*	−0.359*
*P*	.643	.087	.714	.720
Total points				
Control group	100.00 (100.00–100.00)	67.30 ± 11.80	76.17 ± 10.46	92.00 (86.75–94.00)
Observation group	100.00 (100.00–100.00)	74.17 ± 7.78	81.03 ± 8.13	94.00 (89.00–98.00)
*t*/Z	0.452*	4.861†	1.870†	1.925*
*P*	.651	.010	.049	.045

* Z-statistic.

† *t*-statistic.

ADL = ability of daily living, ROM = range of motion.

Repeated-measures analysis of variance revealed significant main effects of time for pain, activities of daily living, range of motion, muscle strength, and total score (*P* < .05), indicating significant changes in these outcomes over time. Significant main effects of the group were observed for pain and total score (*P* < .05), suggesting differences between the groups. However, no significant group differences were found for activities of daily living, range of motion, or muscle strength (*P* > .05). The interaction effect between time and group was not statistically significant for any of the outcomes (*P* > .05), indicating that the changes in outcomes over time did not differ significantly between the 2 groups (Table 3).

**Table 3:** Repeated-measures analysis of variance of CMS between 2 groups.

Item	*F*_intergroup_		*F*_intragroup_		*F*_interaction_	
	*F*	*P*	*F*	*P*	*F*	*P*
Pain	175.118	<.001	5.184	.026	1.634	.183
Activities of daily living	3027.279	<.001	1.794	.186	0.807	.495
Range of motion	584.227	<.001	2.592	.113	1.204	.317
Muscular strength	20.969	<.001	1.535	.220	1.332	.273
Total points	173.610	<.001	6.297	.015	1.572	.206

CMS = Constant–Murley Score.

### 3.2. Comparison of self-efficacy between the 2 groups

Before the intervention, no significant difference was observed in self-efficacy scores between the 2 groups (*P* > .05). However, following the intervention, the self-efficacy score of the intervention group was significantly higher compared to the control group (*P* < .05) (Table 4).

**Table 4:** Intergroup comparison of self-efficacy scores between 2 groups ($\bar{x}$ ± *s*).

Group	Pre-intervention	Post-intervention
Control group	104.63 ± 18.82	106.70 ± 26.11
Observation group	104.77 ± 22.39	124.07 ± 12.87
*t*	0.363	−3.936
*P*	.716	<.001

### 3.3. Comparison of quality of life between the 2 groups

Before the intervention, no significant differences were observed in the scores of each dimension of quality of life between the 2 groups (*P* > .05). However, following the intervention, the intervention group demonstrated significantly higher scores in emotional function, cognitive function, and overall health dimensions compared to the control group (*P* < .05). Conversely, the score of the pain dimension was significantly lower in the intervention group compared to the control group (*P* < .05) (Table 5).

**Table 5:** Intergroup comparison of HRQOL between 2 groups [M (P25, P75)/$\bar{x}$ ± *s*].

Domain		Pre-intervention	Post-intervention
Physiological function	Control group	100.00 (93.33–100)	93.33 (85.00–93.33)
	Observation group	100.00 (66.67–100)	86.67 (85.00–100.00)
	Z	0.422	0.418
	*P*	.673	.676
Role function	Control group	71.67 ± 21.51	75.56 ± 25.42
	Observation group	74.44 ± 20.87	78.89 ± 20.50
	*t*	0.368	0.331
	*P*	.713	.741
Emotional function	Control group	61.67 ± 23.63	78.61 ± 12.51
	Observation group	64.72 ± 23.94	90.28 ± 11.18
	*t*	0.516	3.476
	*P*	.606	.001
Cognitive function	Control group	83.33 (62.50–100.00)	83.33 (66.67–87.50)
	Observation group	91.67 (66.67–100.00)	100.00 (83.33–100.00)
	*Z*	0.776	3.309
	*P*	.438	.001
Social function	Control group	83.33 (66.67–100.00)	100.00 (83.33–100.00)
	Observation group	100.00 (66.67–100.00)	83.33 (83.33–100.00)
	*Z*	1.224	−0.345
	*P*	.221	.730
Overall health status	Control group	66.67 (50.00–83.33)	66.67 (58.33–83.33)
	Observation group	66.67 (56.25–75.00)	83.33 (75.00–83.33)
	*Z*	−0.218	2.954
	*P*	.828	.003
Fatigue	Control group	11.11 (0.00–36.11)	33.33 (22.22–36.11)
	Observation group	11.11 (0.00–22.22)	22.22 (11.11–33.33)
	*Z*	0.488	−0.808
	*P*	.626	.419
Nausea and emesis	Control group	0.00 (0.00–0.00)	0.00 (0.00–0.00)
	Observation group	0.00 (0.00–0.00)	0.00 (0.00–0.00)
	*Z*	−0.582	0.727
	*P*	.561	.467
Pain	Control group	0.00 (0.00–33.33)	16.67 (12.50–33.33)
	Observation group	0.00 (0.00–33.33)	0.00 (0.00–33.33)
	*Z*	0.229	−2.261
	*P*	.819	.024
Dyspnea	Control group	0.00 (0.00–0.00)	16.67 (0.00–33.33)
	Observation group	0.00 (0.00–8.33)	0.00 (0.00–33.33)
	*Z*	0.311	−0.490
	*P*	.756	.624
Sleep disturbances	Control group	0.00 (0.00–33.33)	33.33 (0.00–33.33)
	Observation group	0.00 (0.00–41.67)	0.00 (0.00–33.33)
	*Z*	−0.016	−1.803
	*P*	.987	.071

HRQOL = Health-Related Quality of Life.

## 4. Discussion

In recent years, the prognosis and quality of life of patients with TC have garnered significant attention. It is widely acknowledged that rehabilitation plans and physical interventions play a crucial role in enhancing quality of life and promoting functional recovery in patients with TC, with a particular focus on potential neck complications associated with TC treatment.^[19]^ Comprehensive rehabilitation interventions have been shown to not only improve musculoskeletal strength and reduce systemic inflammatory responses but also alleviate patient symptoms.^[20–22]^ Previous studies have demonstrated that postoperative stretching exercises can mitigate pain and delay dysfunction in patients with TC.^[7,23]^ Additionally, Kim et al^[24]^ have found that family-centered aerobic, resistance, or flexibility exercises can effectively alleviate fatigue symptoms, enhance immune function, and improve postoperative quality of life. Despite growing attention to functional recovery and mental health issues following thyroid tumor surgery, there remains a lack of understanding regarding optimal rehabilitation strategies, both domestically and internationally, with a tendency to overlook patients’ needs and subjective initiative.^[25]^ Furthermore, Goldfarb and Casillas^[26]^ have highlighted the high demand for home rehabilitation information among TC patients, emphasizing the need for nurses to provide corresponding information support. Another study has shown that patients with TC often experience negative emotions such as anxiety and depression, which directly impact their self-efficacy and prognosis.^[27]^ Improving patients’ self-efficacy can effectively alleviate negative emotions in cancer patients, thereby enhancing their rehabilitation compliance. However, at present, there is a paucity of research on the development and implementation of shoulder and neck prevention and rehabilitation nursing programs following TC surgery. Existing intervention methods are relatively simplistic, with a primary focus on medical care, neglecting patient participation as disease managers, and their enthusiasm requires improvement. Therefore, this study aimed to formulate and implement prevention and rehabilitation nursing programs for shoulder and neck discomfort, tailored to patients’ needs, self-efficacy, and cognitive differences, to alleviate their physical and mental burdens.

Empowerment refers to the process of individuals taking control of their affairs, comprising personal ability, natural help systems, active behavior, social policy, and change.^[11]^ In the medical and healthcare field, the empowerment-based intervention model has distinct advantages, including timely identification of clinical and nursing problems, active patient participation in health management, and enhanced personal awareness and disease-coping ability.^[28]^ Currently, empowerment theory is widely applied in the management of chronic diseases, such as diabetes and hypertension, as well as tumor rehabilitation. Stang and Mittelmark^[29]^ confirmed that empowerment intervention during female breast cancer rehabilitation can improve patients’ positive consciousness. Additionally, previous studies^[30]^ have shown that empowerment education not only enhanced the autonomy of cancer patients but also enriched their information support system. This study takes the empowerment theory as its core, developing a detailed nursing plan centered around “patient-centered” care, to meet patients’ needs, dynamically evaluating their rehabilitation and cognitive differences, and improving their self-efficacy. The plan clearly outlines the applicable population, evaluator, perioperative evaluation content, preventive measures, and rehabilitation nursing measures, while also integrating the influencing factors of patients’ shoulder and neck discomfort, providing a comprehensive nursing policy for clinical practice. Following 2 rounds of expert reviews from across the country, the constructed plan was confirmed to be scientific, reliable, and reasonable.

A rational rehabilitation plan or scheme must consider the limitations of individual needs and functions.^[31]^ This study found that the comprehensive scores of shoulders and neck in the intervention group were significantly higher than those in the control group (*P* < .05). A group effect was observed in the pain score and total score through repeated measurement analysis of variance (*P* < .05), indicating that the research scheme effectively improved patients’ discomfort symptoms and enhanced shoulder and neck function. This finding is consistent with previous research.^[32]^ The possible explanation is that patients can dynamically adjust rehabilitation goals and plans according to their actual situation and staged rehabilitation effects after receiving scientific and systematic rehabilitation exercise plans, identify and solve problems while implementing the plans, and ultimately improve their shoulder and neck problems after a period of persistence. Additionally, this study found that the scheme can also improve patients’ self-efficacy and quality of life, further confirming the application value of the empowerment theory-based nursing model in the perioperative period of cancer patients. In the treatment of cancer and its complications, patients’ self-efficacy, emotional state, and coping ability are essential, and the empowerment theory-based nursing model enables patients to feel understood, access better coping resources, and enhance their self-efficacy.^[33]^

This study has several limitations. The small sample size is a major constraint, and the single-center design, where patients were recruited from a single hospital, may limit the generalizability of the findings, requiring further verification to establish the universality of the results.

## 5. Conclusion

In summary, this study adopts a patient-centered approach, developing and implementing a shoulder and neck prevention and rehabilitation program that improves the shoulder and neck function, self-efficacy, and quality of life of patients undergoing TC surgery, thereby demonstrating the clinical application value of the program. However, this study has limitations, including a short intervention period. Future follow-up studies will be conducted to further investigate the long-term effects of this program on shoulder and neck rehabilitation in patients with TC.

## Acknowledgments

The author would like to acknowledge the Zhongshan Hospital affiliated to Xiamen University and all patients for providing data and support.

## Author contributions

**Conceptualization:** Qiuqin Xu.

**Investigation:** Qiuqin Xu.

**Methodology:** Qiuqin Xu, Hongzhan Jiang, Yuanchan Li.

**Software:** Qiuqin Xu, Hongzhan Jiang, Yuanchan Li.

**Writing—original draft:** Qiuqin Xu, Hongzhan Jiang.

**Writing—review & editing:** Xiushan Qi, Lijuan Chen.
